# Antidepressant-induced membrane trafficking regulates blood-brain barrier permeability

**DOI:** 10.1038/s41380-024-02626-1

**Published:** 2024-05-30

**Authors:** Wenjia Du, Huanhuan Chen, Ilona Gróf, Lucien Lemaitre, Alexandra Bocsik, Adrian Perdyan, Jakub Mieczkowski, Mária A. Deli, Tibor Hortobágyi, Qi Wan, Oleg O. Glebov

**Affiliations:** 1 Institute of Neuroregeneration and Neurorehabilitation, Qingdao University, Qingdao, Shandong 266071 China; 2 Institute of Biophysics, HUN-REN Biological Research Centre, Szeged, Hungary; 3 3P-Medicine Laboratory, Medical University of Gdańsk, Gdańsk, 80-210 Poland; 4 Centre for Healthy Brain Ageing, Department of Psychological Medicine, Institute of Psychiatry, Psychology & Neuroscience, King’s College London, De Crespigny Park, London, SE5 8AF UK; 5 Department of Neurology, Faculty of Medicine, University of Debrecen, Debrecen, Hungary

**Keywords:** Neuroscience, Drug discovery, Cell biology

## Abstract

As the most prescribed psychotropic drugs in current medical practice, antidepressant drugs (ADs) of the selective serotonin reuptake inhibitor (SSRI) class represent prime candidates for drug repurposing. The mechanisms underlying their mode of action, however, remain unclear. Here, we show that common SSRIs and selected representatives of other AD classes bidirectionally regulate fluid-phase uptake at therapeutic concentrations and below. We further characterize membrane trafficking induced by a canonical SSRI fluvoxamine to show that it involves enhancement of clathrin-mediated endocytosis, endosomal system, and exocytosis. RNA sequencing analysis showed few fluvoxamine-associated differences, consistent with the effect being independent of gene expression. Fluvoxamine-induced increase in membrane trafficking boosted transcytosis in cell-based blood-brain barrier models, while a single injection of fluvoxamine was sufficient to enable brain accumulation of a fluid-phase fluorescent tracer in vivo. These findings reveal modulation of membrane trafficking by ADs as a possible cellular mechanism of action and indicate their clinical repositioning potential for regulating drug delivery to the brain.

## Introduction

Antidepressant drugs (ADs) are routinely used for treatment of major depressive disorder (MDD), the most common mental health disease worldwide. Since their advent in the 1960s, ADs have remained by far the most common psychotropic drugs, accounting for 83.4 million prescriptions in the year 2021/22 in the UK alone [1]. The majority of currently prescribed ADs belong to the selective serotonin reuptake inhibitor (SSRI) class. Despite the prevalence of SSRIs in the mental health pharmacopeia, the extent of their clinical efficacy against MDD remains unclear [2–4], compounded by the ongoing debate regarding the role of serotonin disbalance in MDD [5–10]. Nevertheless, SSRIs continue to enjoy a remarkably high public profile, not only as MDD therapeutics but also as prime candidates for drug repurposing beyond the sphere of psychiatry, as evidenced in the context of the recent COVID-19 pandemic [11–17].

While SSRIs are generally considered safer and better tolerated than earlier ADs, they are associated with multiple short and long-term side effects [18, 19], affecting adherence [20]. Association between dosage, plasma concentration, clinical efficacy, and side effects of SSRIs remains incompletely understood [21, 22], further obfuscated by their complex metabolism and tissue distribution profile [23]. Lack of knowledge regarding mechanisms of SSRI action across the body continues to hamper their definitive appraisal not only for MDD treatment but also for drug repurposing.

We have previously shown that therapeutic concentrations of selected ADs and other psychotropic drugs can regulate membrane trafficking in non-neuronal cell types [24, 25], raising the possibility that they may control basic cell biological processes across the body. Moreover, recent clinical evidence from several groups including ours has implicated SSRIs in COVID-19 protection and treatment [26–30], suggesting that putative effects of SSRI treatment outside the brain may be of clinical benefit. Here, we show that fluid-phase endocytosis is a key target pathway for concentration-dependent modulation by most SSRIs and some other ADs. Focusing on induction of endocytosis by a canonical SSRI fluvoxamine, we showed that it rapidly upregulated clathrin-mediated pathway in a dynamin-dependent manner, resulting in an increase in endosomal capacity. We further showed that fluvoxamine-regulated membrane trafficking drives transcytosis across the blood-brain barrier in cell-based models and in vivo. Collectively, our findings reveal modulation of membrane trafficking as a nexus for many common psychotropics, suggesting their broad clinical utility for drug delivery applications.

## Results

### Common ADs elicit distinct effects on fluid-phase uptake in the low-therapeutic range of concentrations

**Fig. 1:**
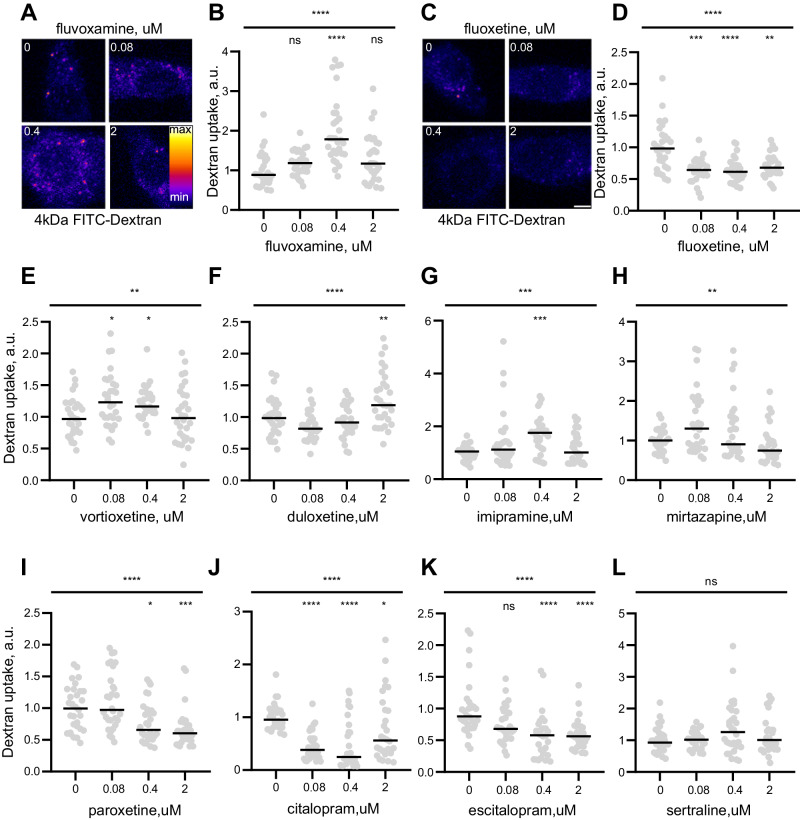
Common ADs bidirectionally modulate fluid-phase endocytosis in the lower-therapeutic concentration range. **A** Representative images of 4 kDa FITC-dextran internalization in PC12 cells treated with fluvoxamine for 1 h. **B** Quantification of dextran internalization in fluvoxamine-treated cells, measured as total fluorescence signal per cell. Values are normalized to control. ****P < 0.0001, Kruskal–Wallis test. ****P < 0.0001, ns–not significant, Dunn’s multiple comparisons test. **C** Representative images of 4 kDa FITC-dextran internalization in PC12 cells treated with fluoxetine for 1 h. **D** Quantification of dextran internalization in fluoxetine-treated cells, measured as total fluorescence signal per cell. Values are normalized to control. ****P < 0.0001, Kruskal–Wallis test.****P < 0.0001, ***P < 0.001, **P < 0.01, Dunn’s multiple comparisons test. Quantification of 4 kDa FITC-dextran internalization in PC12 cells treated with vortioxetine (**E**), duloxetine (**F**), imipramine (**G**) mirtazapine (**H**), paroxetine (**I**), citalopram (**J**), escitalopram (**K**), and sertraline (**L**), measured as total fluorescence signal per cell. ****P < 0.0001, ***P < 0.001, **P < 0.01, *P < 0.05, ns–not significant, Kruskal–Wallis test with Dunn’s multiple comparisons test. N ≥ 3 independent experiments, n = 30–45 cells.Scale bar, 5 μm.

Our previous evidence indicated that a canonical SSRI drug fluvoxamine (brand names Faverin® and Luvox®) regulates fluid phase uptake and coronavirus Spike protein endocytosis in human embryonic kidney 293 cells [25]. To confirm this observation, we applied a low-therapeutic range of concentrations (80 nM–2 μM) of fluvoxamine to the rat pheochromocytoma 12 (PC12) cell line which is routinely used in neuroscience and pharmacology studies [31]. In concord with the earlier findings, 1 h incubation with fluvoxamine led to the increased uptake of a fluid phase marker 4 kDa dextran labeled with fluorescein isothiocyanate (FITC), henceforth referred to as 4 kDa FITC-dextran (Figs. 1A, B and S1A). Interestingly, the effect was most pronounced at 400 nM, which corresponds to the average plasma concentration of fluvoxamine in MDD patients [32]. Unexpectedly, another common SSRI fluoxetine (Prozac®) had the opposite effect, blocking fluid-phase uptake of 4 kDa FITC-dextran at concentrations as little as 80 nM (Figs. 1C, D, and S1B). Neither drug affected cell viability following 1 h of treatment (Fig. S1C, D).

We then used the 4 kDa FITC-dextran uptake assay to test the effects of 5 other SSRIs currently used for MDD treatment in the UK [33], namely sertraline (Zoloft®), citalopram (Celexa®), escitalopram (Cipralex®), paroxetine (Paxil®, Seroxat®), and vortioxetine (Trintellix®). Additionally, we investigated several drugs representing other AD classes, including serotonin and norepinephrine reuptake inhibitor (SNRI) duloxetine (Cymbalta®), atypical antidepressant mirtazapine (Remeron®), and a canonical tricyclic AD imipramine (Tofranil®). Remarkably, we found that most of the tested drugs affected membrane trafficking when applied in the 80 nM–2 µM range of concentrations (Figs. 1E–L, and S1E–H). Vortioxetine, imipramine, mirtazapine, and duloxetine all increased fluid phase uptake in a concentration-dependent manner, similar to the effect of fluvoxamine (Figs. 1E–H, and S1E–H). Conversely, treatment with citalopram, escitalopram, and paroxetine resulted in a significantly diminished dextran uptake akin to the effect of fluoxetine (Figs. 1I–K, and S1I–L), while sertraline had no significant effect (Figs. 1L, and S1J). Thus, diverse ADs can bidirectionally regulate membrane trafficking at therapeutically relevant concentrations or below.

### Fluvoxamine enhances endosomes and induces clathrin-mediated endocytosis

We decided to investigate the mechanism of fluvoxamine-induced endocytosis in more detail due to its emerging relevance for drug repurposing in COVID-19 and beyond [12, 25, 28–30, 34–36]. Fluvoxamine-induced increase in fluid-phase endocytosis was confirmed using another fluorescent fluid-phase marker, a negatively charged hydrophilic dye Lucifer yellow (Fig. S1M, N), suggesting that the effect was not limited to dextran uptake. In the light of our previous data showing that glutamate receptor antagonists may regulate the endosomal system [24], we sought to assess the effect of fluvoxamine on endosomes.

**Fig. 2:**
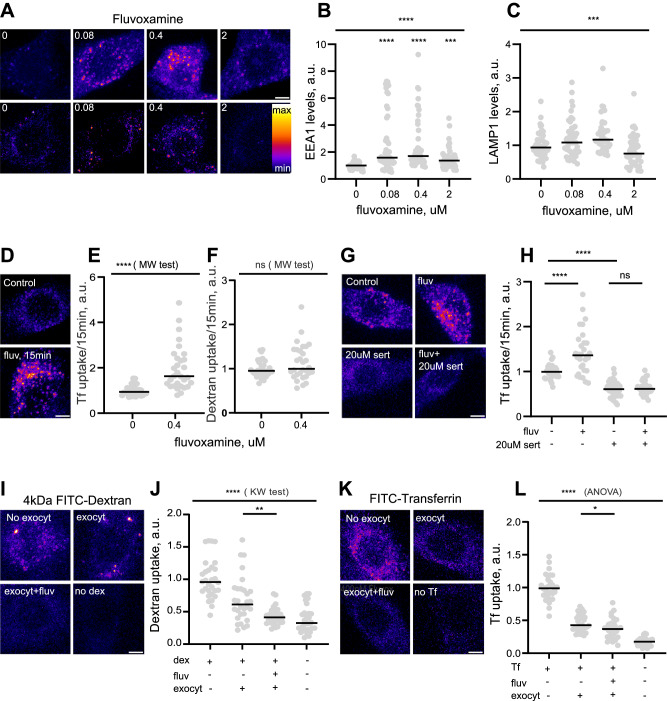
Fluvoxamine rapidly enhances endosomal network capacity, clathrin-mediated endocytosis, and exocytosis. **A** Representative image of PC12 cells treated with fluvoxamine for 1 h and immunostained for EEA1 and LAMP1. **B** Quantification of EEA1 levels in fluvoxamine-treated cells. Values are normalized to control. ****P < 0.0001, ****P < 0.0001, ***P < 0.001, Kruskal–Wallis test with Dunn’s multiple comparisons test. **C**. Quantification of LAMP1 levels in fluvoxamine-treated cells. ***P < 0.001, ns-not significant, Kruskal–Wallis test with Dunn’s multiple comparisons test. **D** Representative images of Tf internalization in PC12 cells treated with fluvoxamine for 15 min. **E** Quantification of Tf internalization in cells treated with fluvoxamine for 15 min. ****P < 0.0001, Mann–Whitney test. **F** Quantification of 4 kDa FITC-dextran internalization in PC12 cells treated with fluvoxamine for 15 min. ns–not significant, Mann–Whitney test. **G** Representative images of Tf internalization in PC12 cells incubated with 400 nM fluvoxamine and 20 µM sertraline for 15 min. **H** Quantification of Tf internalization in cells treated with fluvoxamine and 20 µM sertraline. ****P < 0.0001, ns–not significant, Kruskal–Wallis test with Dunn’s multiple comparisons test. **I** Representative images of 4 kDa FITC-dextran exocytosis in PC12 cells treated with fluvoxamine. **J** Quantification of the effect of fluvoxamine on 4 kDa FITC-dextran exocytosis. ****P < 0.0001, **P < 0.01, Kruskal–Wallis test with Dunn’s multiple comparisons test. **K** Representative images of FITC-transferrin exocytosis in PC12 cells treated with fluvoxamine. **L** Quantification of the effect of fluvoxamine on FITC-transferrin exocytosis. ****P < 0.0001, *P < 0.05, 1-way ANOVA with Dunn’s multiple comparisons test. N ≥ 3 independent experiments, n = 30–45 cells. Scale bar, 5 μm.

We immunostained PC12 cells for the canonical endosomal marker proteins early endosomal antigen 1 (EEA1) and lysosomal-associated marker protein 1 (LAMP1), labeling early and late endosomes respectively. Fluvoxamine treatment strongly upregulated early endosomes and modestly enhanced late endosomes as evidenced by EEA1 and LAMP1 labeling (Fig. 2A–C). Conversely, fluoxetine treatment modestly diminished EEA1 and LAMP1 staining at 400 nM concentration (Fig. S2A–C). Taken together, these findings suggest that fluvoxamine increases the capacity of the endosomal system in PC12 cells in a concentration-dependent manner.

We further sought to identify the endocytic pathway regulated by fluvoxamine. The key pathway involved in fluid-phase uptake in PC12 cells is clathrin-mediated endocytosis (CME) [37]. To assess the effect of fluvoxamine on CME, we incubated cells with FITC-labeled transferrin (Tf), which upon binding to its receptor is rapidly and selectively internalized via receptor-mediated endocytosis in a clathrin-dependent manner [38]. Treatment with fluvoxamine for as little as 15 min significantly increased Tf endocytosis while having no significant effect on 4 kDa FITC-dextran uptake, indicating rapid CME induction by fluvoxamine (Fig. 2D–F). To further confirm the effect of fluvoxamine on CME, we leveraged the previously reported inhibitory effect of higher-than-therapeutic concentration of sertraline on a large GTPase dynamin, a key driver of CME [39]. While treatment for 1 h with 20 µM sertraline resulted in significant cell detachment, confirming inhibition of CME [40], a shorter 15 min treatment did not visibly affect cell numbers yet effectively blocked transferrin internalization in both control and fluvoxamine-treated cells (Fig. 2G, H). These results demonstrate that fluvoxamine rapidly upregulates dynamin-dependent CME in PC12 cells.

Besides CME, another endocytic pathway involved in fluid-phase uptake in PC12 cells is macropinocytosis, involving cytoskeleton-regulated remodeling of the plasma membrane. To investigate the effect of fluvoxamine on macropinocytosis, we visualized internalization of 70 kDa dextran labeled with tetramethylrhodamine (TRITC) which represents a selective marker for macropinocytosis, in contrast to non-selective fluid-phase uptake of 4 kDa dextran [41]. Fluvoxamine treatment resulted in a significant decrease in intracellular TRITC signal, consistent with reduced internalization of 70 kDa dextran (Fig. S2D, E). A similar result was observed with uptake of 2 MDa dextran, although in this case intracellular accumulation was barely observed due to its high molecular weight (Fig. S2F, G). Application of 5-(n-ethyl-n-isopropyl)-amiloride (EIPA) prevented fluvoxamine-induced increase in 4 kDa dextran internalization, consistent with the previously reported EIPA effect on both macropinocytosis and receptor-mediated endocytosis [42, 43] (Fig. S2H, I). Taken together, these observations are consistent with specific upregulation of CME and downregulation of macropinocytosis by fluvoxamine.

### Fluvoxamine-induced exocytosis to match endocytosis

To maintain cell integrity, removal of plasma membrane material from the cell surface through endocytosis must be coordinated with membrane recycling through exocytosis. Our previous data showed that surface levels of angiotensin-converting enzyme 2—the key cell surface receptor for COVID-19 infection—remained stable despite fluvoxamine-induced Spike protein internalization, implying that endocytic rate increase was likely matched by enhanced exocytosis [25].

To directly visualize the effect of fluvoxamine treatment on exocytosis, we measured loss of intracellular fluorescence from cells preloaded with 4 kDa FITC-dextran for 1 h. Treatment with fluvoxamine led to a significantly accelerated loss of fluorescence within 20 min of treatment compared to vehicle-treated cells, indicating that exocytosis of intracellular fluid-phase material back to the extracellular milieu was rapidly enhanced by fluvoxamine (Fig. 2I, J). Similar results were obtained for transferrin (Fig. 2K, L). Taken together, these data show that fluvoxamine enhances exocytosis as well as endocytosis, consistent with lack of visible morphological changes following low-dose fluvoxamine treatment [25].

### Analysis of fluvoxamine effect on gene expression

Previous reports suggest that fluvoxamine treatment may have an effect on gene expression [44], including that of transcription factors involved in cell signaling e.g., Notch1 [45]. To investigate whether expression of membrane trafficking machinery was affected by various concentrations of fluvoxamine, we used next-generation RNA sequencing (RNA-seq) to analyze the transcriptome of PC12 cells following 1 h treatment with 80 nM, 400 nM, and 2 µM. Since there is no consensus on the best RNA-seq pipeline, fastq files were processed using two separate alignment tools to minimize potential bias [46].

**Fig. 3:**
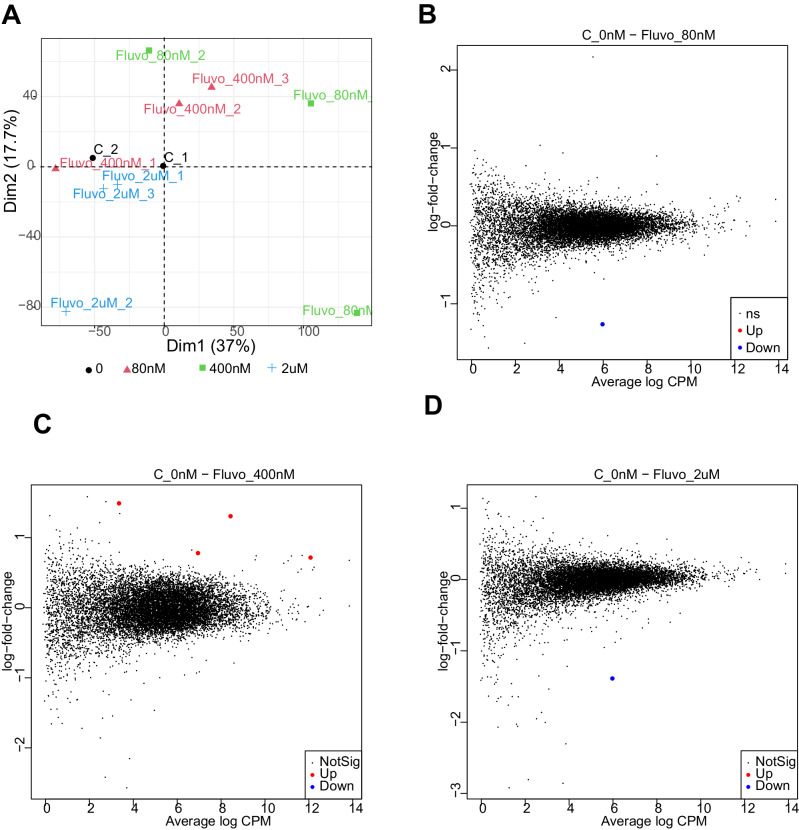
RNAseq analysis of fluvoxamine effect shows concentration-specific effects on gene expression. **A** Principal component analysis of all replicates, Dimensions 1–2. **B** Mean difference plot for gene expression between control and 80 nM fluvoxamine. CPM, count per million. **C** Mean difference plot for gene expression between control and 400 nM fluvoxamine. CPM, count per million. **D** Mean difference plot for gene expression between control and 2 μM fluvoxamine. CPM, count per million.

In both approaches, principal component analysis and dendrogram plots showed a major overlap between all samples (Figs. 3A, and S3A, B). Only a few differentially expressed genes were found to fulfill established thresholds of significance (Fig. 3B–D, Table S1). Interestingly, the only mRNA induced by more than one concentration of fluvoxamine was that encoding early growth receptor 1 (Egr1) (Table S1), a key transcription factor regulating activity-dependent transcription in the brain, hinting at possible longer-term effects of fluvoxamine on gene expression [47]. Nevertheless, these data clearly showed that the rapid effect of fluvoxamine on endo/exocytosis is likely unrelated to gene expression, more likely arising from direct modulation of the cell biology mechanisms.

### Fluvoxamine increases transcytosis in brain endothelial cells

Brain microvascular endothelial cells are the main component of the blood-brain barrier. On the basis of our observations in cell cultures, we hypothesized that fluvoxamine induces transcytosis across the blood-brain barrier in vivo, thereby increasing accessibility of the brain to macromolecular cargo. To test this hypothesis, we measured the effect of fluvoxamine on penetration of 4 kDa FITC-dextran into the brain in adult mice.

**Fig. 4:**
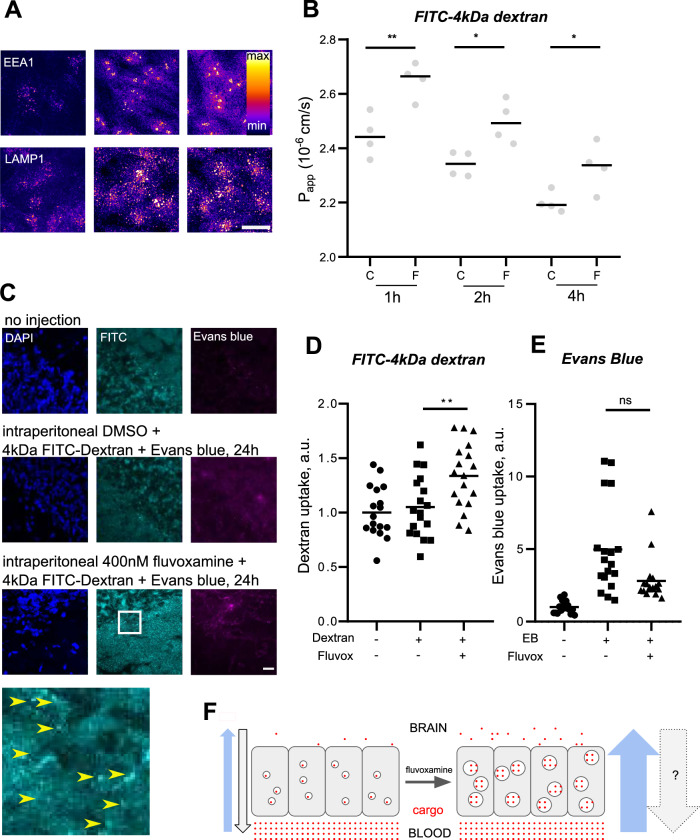
Fluvoxamine-induced transcytosis boosts permeability of the blood-brain barrier. **A** Representative image of cultured brain-like endothelial cells treated with fluvoxamine for 1 h and immunostained for EEA1 and LAMP1. Scale bar, 20 µm. **B** Permeability of 4 kDa FITC-dextran across the human co-culture model of the blood-brain barrier in the presence of 80 nM fluvoxamine after 1, 2, and 4 h of incubation. Values presented are means ± SD. **P < 0.01, *P < 0.05, two-way ANOVA, with Bonferroni posttest; n = 4. **C** Representative images of brain sections from mice injected intraperitoneally 24 h prior with 4 kDa FITC-dextran, Evans blue, and fluvoxamine. Arrows denote accumulation of dextran in multiple punctate intracellular structures. Scale bar, 20 µm. **D** Quantification of FITC signal in brain sections from mice injected intraperitoneally 24 h prior with 4 kDa FITC-dextran, Evans blue, and fluvoxamine. **P < 0.01, One-way ANOVA with Tukey’s post test. N = 3 independent experiments, 9 animals/condition. **E** Quantification of Evans Blue signal in brain sections from fluvoxamine-treated mice. ns - *P* = 0.23, Kruskal–Wallis test with Dunn’s multiple comparisons test. **F** Schematic model of fluvoxamine-induced transcytotic dextran delivery into the brain. Under normal conditions (left) transcytosis in the blood-brain barrier cells is inactive, limiting delivery of cargo from the bloodstream into the brain milieu. Treatment with the therapeutic concentration of fluvoxamine (right) triggers fluid-phase transcytosis across the blood-brain barrier, resulting in enhanced delivery of non-charged hydrophilic cargo. Blue arrows denote fluvoxamine-induced bloodstream-to-brain flow of blood-brain barrier transcytosis; gray arrows denote speculated compensatory increase in the brain-to-bloodstream flow. For the sake of simplicity, putative effects of fluvoxamine on uptake of negatively charged cargo are not pictured.

Real-time measurement showed that up to 1 µM concentration of fluvoxamine did not affect cell layer impedance, indicating that integrity of the monolayer was unaffected (Fig. S4A, B). Consistent with the observations in PC12 cells, fluvoxamine treatment of human brain endothelial cells at 80 or 400 nM concentration enhanced punctate labeling for EEA1 and LAMP1, indicative of enhanced endosomal network (Fig. 4A). Fluvoxamine increased internalization of Lucifer yellow after 4 h (Fig. S4C) but had no effect on internalization of galectin-1 labeled by a fluorescent dye Atto 488 (Fig. S4D).

To analyze the effect of fluvoxamine on permeability of the blood-brain barrier in a cell-based system, we used an insert-based co-culture of endothelial cells with brain pericytes previously established by us [48]. This model reliably recapitulates the key aspects of brain microvessel endothelium such the expression of tight junctions and other blood-brain barrier characteristics, including apico-basal polarization [48, 49]. The permeability of 4 kDa FITC-dextran across the co-culture model of the blood-brain barrier was significantly increased by fluvoxamine treatment at the 1-, 2- and 4-h time-points (Fig. 4B). Fluvoxamine also increased the permeability of endogenous galectin-1 (15 kDa) across the blood-brain barrier model at the 2-h time-point compared to the control groups (Fig. S4E). Conversely, the penetration of the albumin-Evans blue complex (67 kDa) across the blood-brain barrier co-culture model was not altered by fluvoxamine (Fig. S4F). Taken together, these data are consistent with fluvoxamine inducing permeability of the human brain endothelial cells through transcytosis.

### Fluvoxamine injection regulates blood-brain barrier permeability in vivo

Brain microvascular endothelial cells are the main component of the blood-brain barrier. On the basis of our observations in cell cultures, we hypothesized that fluvoxamine induces transcytosis across the blood-brain barrier in vivo, thereby increasing accessibility of the brain to macromolecular cargo. To test this hypothesis, we measured the effect of fluvoxamine on penetration of 4 kDa FITC-dextran into the brain in adult mice.

While 4 kDa FITC-dextran was undetectable in vehicle-treated brains, fluvoxamine-induced accumulation was evident as soon as 1 h following intravenous co-injection of dextran and fluvoxamine (Fig. S4G), consistent with a rapid increase in blood-brain barrier permeability to the fluid-phase marker. To investigate the longer-term systemic effect of fluvoxamine application, we injected fluvoxamine and dextran intraperitoneally and measured the intensity of FITC signal in the brain 24 h later. Again, fluorescence levels in vehicle-treated animals indicated negligible dextran penetration across the blood-brain barrier, while co-injection of 400 nM fluvoxamine resulted in notable accumulation of FITC signal in the brain tissue (Fig. 4C, D). FITC signal was accumulated in punctate structures across the brain tissue, reminiscent of observed endosomal accumulation of dextran in PC12 cells and consistent with increased membrane trafficking across the blood-brain barrier (Fig. 4C, magnified panel). Conversely, animals in the control group exhibited diffuse accumulation of Evans blue signal across the brain tissues, consistent with extravasation of albumin-Evans blue complex, which was reduced by fluvoxamine (Fig. 4C, E). Taken together, these results demonstrate that fluvoxamine-induced transcytosis increases the permeability of the blood-brain barrier in vivo for 4 kDa dextran, while restricting ingress of albumin-Evans blue complex [50].

## Discussion

Despite the long-term prominence of ADs and particularly SSRIs in psychiatry, the mode of their therapeutic action is hotly debated to this day [5–10], and the mechanisms underlying their side effects remain unknown. In this study, we provide key evidence filling this knowledge gap by showing that most if not all of currently prescribed SSRIs are capable of rapidly regulating membrane trafficking. We further identify the pathway regulated by fluvoxamine and demonstrate its functional significance in blood-brain barrier permeability. Considering the myriad of roles for membrane trafficking in health and disease [51] and the high profile of ADs, our findings are likely to have significant ramifications for understanding their mode of action and side effects, as well as for re-appraisal of their therapeutic potential.

The focus on the therapeutically relevant concentration range has been a key consideration in our work, setting it apart from earlier mechanistic studies of AD cell biology. While blood plasma levels of ADs in MDD patients can vary between 30 nM and 3.75 µM [52, 53], the overwhelming majority of cell-based studies to date employed 10- to 10^3^-fold higher concentrations. Such amounts of ADs tend to evoke pleiotropic effects, ranging from disruption of lipid homeostasis [13], inhibition of acid sphingomyelinase [54], direct binding to neurotrophin receptors [55], to blockade of membrane trafficking [39, 56], as well as widespread cytotoxicity [13, 57, 58]. In contrast, our study has focused on the lower end of therapeutic concentration range, which is characterized by high tolerability and strong dose-response relationship in the clinic [21]. In the light of the emerging importance of therapeutic drug monitoring for balancing AD efficacy and side-effect burden [59, 60], future investigation of concentration-dependent SSRI action will need to consider complexities of their tissue distribution, metabolism, and clearance [23, 61, 62].

While the molecular mechanism for the SSRI effect on membrane trafficking remains unclear, most of previously suggested mechanisms can be ruled out logically. The opposite effects of different SSRIs and similar effects of non-SSRI ADs (Figs. 1, and S1) argue against the role for serotonin transporter blockade [18]. Functional inhibition of acid sphingomyelinase [54] would be expected to elicit similar effects from all tested drugs, while activation of sigma-1 receptors [63] would entail similar outcomes from fluoxetine and fluvoxamine treatment; our evidence suggests that neither is the case (Figs. 1, and S1). Other recently suggested mechanisms of AD action involving lipid accumulation and neurotrophin signaling [13, 55] can also be discounted on the basis of their requirement of micromolar drug concentrations, 1–2 orders of magnitude beyond the therapeutic range used in our study.

One possible candidate mechanism for membrane trafficking regulation by ADs is direct concentration-dependent interaction with the membrane itself [64–67]. While there was no obvious association between basic physico-chemical features of ADs and their effect on endocytosis (Table S2), recent evidence points at their differential distribution in biomembranes, consistent with the canonical notion of membrane heterogeneity [68, 69]. It is therefore conceivable that differential partitioning into membranes, or leaflets of the same membrane, or even laterally heterogeneous domains of the same leaflet of the same membrane, may explain at least some of the diverse AD effects on membrane trafficking reported here.

The notion of direct AD-biomembrane interaction is consistent with the rapid timescale of the fluvoxamine effect (Fig. 2) and lack of differential gene expression (Fig. 3). Moreover, changes in membrane properties could differentially affect transport of neutral and charged cargoes in vivo, as evidenced by the effect of fluvoxamine on uptake of FITC-dextran *vs* albumin-Evans blue complex (Fig. 4). It will be of interest to see how this regulatory mechanism fits alongside others, including modulation of surface charge for paracellular transport [67] and gene expression induced by fluid flow [48]. At any rate, in-depth mechanistic investigation of drug-membrane-cargo interaction in SSRI-dependent membrane trafficking goes beyond the scope of this study and will necessarily rely on computational molecular modeling and experimentation involving defined model membrane systems.

One particularly intriguing if speculative possibility arising from our findings is a potential alternative mechanism for AD action in MDD by regulation of serotonin transport across the blood-brain barrier. While the blood-brain barrier is normally impermeable to serotonin, with 10^3^–10^4^ fold lower brain levels compared to the plasma [70], modulation of blood-brain barrier permeability by ADs may affect brain serotonin levels. It must be emphasized that experimental testing of this hypothesis will rely on rigorous measurement of serotonin transport between the body and the brain.

AD-dependent membrane trafficking modulation opens up significant opportunities for improving drug delivery across the blood-brain barrier for treatment of brain disease, e.g., tumors and neurodegenerative disorders [71–73]. While drug development in recent years has excelled in designing biologics such as antibodies, their brain penetration during systemic administration is very low, necessitating high dosages that drive up the cost of treatment and likelihood of adverse effects [74–76]. AD-modulated membrane trafficking may help address this challenge by leveraging fluvoxamine-aided transcytosis to boost transport across the blood-brain barrier (Fig. 4). Notwithstanding the possible risks associated with brain ingress of undesirable drugs, the largely non-specific nature of fluvoxamine-induced transport may obviate the need for complex, expensive and time-consuming modification of biologics [74], while simultaneously eluding the safety concerns associated with blood-brain barrier disruption e.g., by ultrasound [77]. In the longer term, wide-scale modulation of membrane trafficking by these cheap, safe, and well-characterized drugs may provide a conceptually novel method for universal drug delivery, warranting further investigation in relevant disease models as well as in the clinic.

## Materials and methods

See Supplementary Materials.

## Supplementary information

Supplemental material

## Data Availability

Data is available from the authors upon written request.
