# Supplementary material for: Antidepressant-induced membrane trafficking regulates blood-brain barrier permeability
Source: Mol Psychiatry. 2024 May 30;29(11):3590–8. doi: 10.1038/s41380-024-02626-1 (PMC11541205; doi:10.1038/s41380-024-02626-1)
Supplement: Supplementary file 1 — Supplemental material [file 41380_2024_2626_MOESM1_ESM.pdf]

Supplementary materials

Materials & Methods

Reagents, drugs, fluorescent markers

Cell culture materials were from Solarbio and Procell. C57 BL/6 mice were from Jinan Pengyue Laboratory Animal Co. ADs (fluvoxamine, fluoxetine, vortioxetine, duloxetine, imipramine, mirtazapine and paroxetine, citalopram, escitalopram and sertraline) were from AbMole. 4 kDa FITC-dextran was from Sigma-Aldrich. 70 kDa TRITC-dextran was from Chondrex Inc. 2MDa FITC-Dextran was from Sigma-Aldrich. FITC-transferrin was from Jackson Immunoresearch. Lucifer yellow was from Santa Cruz. EIPA was from Selleck. Evans blue was from Solarbio. TRIzol Reagent was from Invitrogen.

Cell culture

PC12 cells were grown and routinely maintained in high-glucose Dulbecco's modified Eagle medium (DMEM) (Solarbio) supplemented with 10% fetal bovine serum FBS (Procell) and 1% penicillin/streptomycin (Gibco) at 37°C in a humidified incubator with 5% CO2 with 10% bovine serum. For the cellular uptake experiments and Immunocytochemical experiments, PC12 cells were cultured in 24-well plates (3×10^4 cells/well, Corning Costar).

Human vascular endothelial cells were cultured in endothelial cell culture medium (ECM-NG, Sciencell, Carlsbad, CA, USA) supplemented with 5% fetal bovine serum (FBS), 1% endothelial growth supplement (Sciencell, California) and 0.5% gentamicin as described in our previous publications (Santa-Maria et al, 2021). Cells were used at passage 7. Bovine brain pericytes (PC) were seeded (≤ P15) into 0.2% gelatin coated dishes (Corning Costar Co., MA, USA) in DMEM (Life Technologies, Thermo Fisher Scientific, USA) supplemented with 20% FBS, 1% Glutamax (Life Technologies) and 0.5% gentamicin (Santa-Maria et al, 2021). For the cellular uptake experiments, human endothelial cells were cultured in 24-well plates (3×10^4 cells/well, Corning Costar) coated with collagen type IV (100 µg/ml) in culture medium supplemented with pericyte-conditioned medium (1:1).

Membrane trafficking assays

All uptake experiments were performed at 37°C. For the uptake experiments, we used fluorescently labeled dextran with different sizes of molecular weight (4 kDa, 70 kDa, 2 MDa), fluorescently labeled transferrin, and Lucifer yellow. Stock solutions of the above prepared in dimethylsulfoxide (DMSO) were diluted in the culture medium to the appropriate concentration, then added to the coverslips in presence of indicated concentrations of drugs or DMSO alone for indicated periods of time. After uptake, the cells were fixed with 4% paraformaldehyde (PFA) and washed six times with phosphate-buffered saline (PBS). Coverslips were then mounted in Fluoromount-G mounting medium (Southern) and stored at 4°C until imaging. 4 kDa FITC-dextran was used at the final concentration of 1 mg/ml, 70 kDa TRITC-dextran at 1 mg/ml, 2 MDa FITC-Dextran at 2 mg/ml, Tf at 5 µg/ml, Lucifer yellow at 1

mg/ml. EIPA concentration used for blockade of macropinocytosis was 25 μM, and sertraline concentration for blockade of CME was 20 μM.

To measure exocytosis, 4 kDa FITC-dextran was added to the culture medium to the final concentration of 1 mg/ml. Cells were washed three times with 500 μl of empty medium, then incubated with FITC-dextran-containing medium for 1 hour at 37°C. Following this, cells were taken out of the incubator, washed three times with 500 μl of culture medium, then incubated with the medium containing 400 nM fluvoxamine or DMSO alone for 20 min at 37°C. Following this, cells were washed, fixed with 4% PFA, and the coverslips were then mounted in Fluoromount-G and stored at 4°C until imaging. Percentage of fluorescence signal loss was used as a readout for exocytosis. Same experimental steps and conditions were used for analysis of Tf exocytosis.

Blood-brain barrier measurements *in vitro*

Fluvoxamine was diluted from 10 mM stock solutions in DMSO into cell culture medium or phenol red-free DMEM/HAM's F-12 medium (Gibco) supplemented with 1% FBS. The final concentration of fluvoxamine was 80 nM for the uptake and permeability assays. In the uptake assay the following concentrations of the fluorescent markers were used: Lucifer yellow, 500 μM; Atto488 labeled galectin-1, 500 nM. In the permeability assay the penetration of 4 kDa FITC-dextran (100 μg/ml), Evans-blue labeled albumin (EBA; 67 kDa; 10 mg/ml bovine serum albumin (BSA) + 167.5 μg/ml Evans-blue) and Atto488-galectin-1 (15.5 kDa; 500 nM) were examined.

The effect of fluvoxamine on the viability of human endothelial cells was monitored by label-free, non-invasive impedance measurements using a real-time cell analysis single plate system (Agilent). Cells were seeded at a density of 6 × 10^3 cells/well to 96-well plate with integrated gold electrodes (E-plate 96, ACEA Biosciences) coated with 0.2% gelatin. To differentiate the cells, culture medium was supplemented with 50% pericyte-conditioned medium. Confluent layers of human brain endothelial cells were treated in the plateau phase with 30 nM, 100 nM, 300 nM, 1000 nM concentrations of fluvoxamine. Triton X-100 (1 mg/ml) was used as a reference compound to induce cell toxicity.

For measurements of endocytic uptake, human endothelial cells were cultured in 24-well plates (3 × 10^4 cells/well, Corning Costar) coated with collagen type IV (100 μg/ml). The culture medium was supplemented with 50% pericyte-conditioned medium. Confluent monolayers of human brain endothelial cells were incubated with Lucifer yellow and Atto488-galectin-1 in the presence of fluvoxamine at 37 °C for 1, 2 or 4 hours. After treatment, cells were washed three times with ice-cold PBS supplemented with 0.1% BSA, once with acid stripping buffer (glycine 50 mM, NaCl 100 mM, pH 3) to remove cell surface-associated fluorescent marker molecules, and finally again with PBS. At the end of the experiments, cells were lysed in distilled water containing 1% Triton X-100. The fluorescent signal of Lucifer yellow (excitation: 420 nm, emission: 535 nm) and Atto488-galectin-1 (excitation: 502 nm, emission: 525 nm) was quantified from the cell lysate by spectrofluorometry (Fluorolog 3, Horiba Jobin Yvon). The level of internalized markers was normalized to the protein content of the cell lysates as measured by BCA Protein Assay (Thermo Fisher).

To establish a cell-based blood-brain barrier model, human endothelial cells and brain pericytes were co-cultured for 6 days on 0.4 μm pore size 0.3 cm^2 Transwell inserts (Corning

Costar Co.) placed in 24-well plates (Santa-Maria et al. 2021). Brain pericytes were plated at 1.8×10^4 cells/cm^2 onto culture inserts coated with collagen type IV (100 µg/ml) and incubated for 3 hours at 37 °C. Human endothelial cells were seeded to the upper side of the membranes (4×10^4 cells/cm^2) which were coated with collagen type IV (100 µg/ml) and fibronectin (25 µg/ml). During co-culture both compartments were filled with endothelial medium. Cells were considered ready for experimentation once the trans-endothelial electrical resistance values have stabilized.

For measurements of permeability, the inserts were transferred to 24-well plates containing 900 µl phenol red-free DMEM/HAM's F-12 medium supplemented with 1% FBS in the acceptor (abluminal) compartments. In the donor (luminal) compartments 200 µl medium was pipetted containing 4 kDa FITC-dextran and Evans blue-albumin, or galectin-1-Atto488 in the presence of fluvoxamine. To avoid unstirred water layer effect, the plates were kept on a horizontal shaker (120 rpm) during the assay. Permeability was measured after 1, 2, and 4 hours. Samples were collected from both compartments and the fluorescent signal of 4 kDa FITC-dextran (excitation: 485 nm; emission: 515 nm), Evans blue-albumin (excitation: 584 nm; emission: 663 nm), and Atto488-galectin-1 (excitation: 502 nm, emission: 525 nm) was quantified with a spectrofluorometer (Fluorolog 3, Horiba Jobin Yvon). Concentrations of each marker were calculated by calibration curves. To determine the apparent permeability coefficient (P_{app}) the clearance (µl) was calculated with the help of the following equation:

$$Cl = \frac{[C]_{ab} \times V_{ab}}{C_l}$$

where Cl is the clearance, $[C]_{ab}$ and V_{ab} represent the concentration and volume (µl) of the abluminal (acceptor) compartment, and $[C]_l$ represents the luminal (donor) concentration. Cumulative clearance values of the marker molecules were plotted against time, and the slope values were used as PS (permeability surface area product). The P_{app} of the markers was calculated from the following equation:

$$P_{app} = \frac{PS}{A}$$

where PS was expressed as clearance rate (µl/min) while A was the surface area of the membrane (0.3 cm^2).

Blood-brain barrier measurements *in vivo*

For measurement of short-term blood-brain barrier permeability. C57 BL/6 mice (8-10 weeks old, male, weighing 22-25 g) were randomly allocated into the drug treatment, vehicle and negative control groups. Fluvoxamine and 4 kDa FITC-Dextran were dissolved in PBS and injected into the tail vein to reach final concentrations of 80 nM 400 nM and 2 µM for fluvoxamine, and 10mg/ml for FITC-dextran, respectively. After 1 h, animals were sacrificed, brains were fixed with 4% PFA, cut into into 30 µm-thick sections with a frozen slicer, mounted onto positively-charged microscope slides in media containing 4′,6-diamidino-2-phenylindole (Solarbio), and imaged using a confocal microscope.

For measurement of longer-term blood-brain barrier permeability, mice were treated as above. Fluvoxamine and 4 kDa FITC-Dextran were dissolved in PBS and injected intraperitoneally to reach final concentrations of 400 nM for fluvoxamine and 10mg/ml for FITC-dextran. Evans

blue was administered as a 2% solution at 4 ml/kg body weight. After 24 hours, animals were sacrificed, and the brains processed as above.

Immunocytochemistry

All steps were performed at room temperature. Coverslips were fixed with 4% PFA in PBS for 20 min and permeabilized in 0.3% Triton-X100 in PBS supplemented with 5% sheep serum for 30 min. Subsequent incubations were carried out in the permeabilization buffer. Coverslips were incubated with appropriate primary antibodies for 1 h, washed three times in PBS and incubated with AlexaFluor-488 and AlexaFluor-594-conjugated secondary antibodies for 1h. Coverslips were then mounted in Fluoromount-G and stored at 4°C until imaging. Antibodies used in the study are listed in **Table S3**.

Confocal microscopy imaging

Samples were imaged on Nikon Eclipse Ti2 laser confocal microscopes equipped with a standard set of lasers and objectives. The imaging systems were controlled by the NIS Elements 2.0 software. The imaging parameters for Nikon Eclipse Ti2 were as follows. The following image acquisition settings were used for serial confocal z-stack images: cells - 1.5 µm step, 512 x 512 pixels, 1x zoom, 100x magnification; tissue sections - 0.5 µm step (short-term assay) or 1.5 µm (long-term assay) 512 x 512 pixels, 2x zoom, 40x magnification. Pinhole size was kept to 1-2 Airy units. Excitation laser wavelengths were 488 nm and 561 nm. Bandpass filters were set at 500−550 nm (FITC, AlexaFluor488) and 570−620 nm (TRITC, AlexaFluor594). Gain, exposure and offset settings were optimized within each experiment to ensure appropriate dynamic range, low background and optimal signal/noise ratio.

RNA-seq and gene expression analysis

PC12 cells were treated with fluvoxamine at 80 nM, 400 nM or 2 µM for 1h. Total RNA was extracted by TRIzol Reagent. RNA sequencing and quality control were provided by Beijing Novogene. Raw data (raw reads) of fastq format files were processed removing reads containing adapter, reads containing poly-N and low-quality reads from raw data. Hence, all the downstream analyses were based on the clean high-quality fastq files. Reference genome and gene model annotation files were downloaded from Ensembl genome database project (www.ensembl.org). Reads alignment was done twice using Hisat2 version 2.1.0 with feature Counts version 2.0.0 for quantification of gene expression and STAR aligner separately. Further, we filtered established count matrices to include protein coding genes with standard deviation > 0 when comparing each experimental arm to control. Additionally, we included genes in which minimum number of samples and minimum expression (count per million) were >=5 and >=1, respectively. To establish research models for differential expression analysis, we built the EdgeR object using limma package (1). We normalized RNA-sequencing data using weighted trimmed average of the log expression ratios, and performed differential gene analysis for pair comparisons fitting a negative binomial generalized log-linear model with applied thresholds of false discovery rate (FDR) <0.05. Downstream analysis was done in the R Studio software (http://www.rstudio.com). PCA plots and hierarchical clustering were done using the factoextra package (https://rpkgs.datanovia.com/factoextra/index.html). The R code used in the RNA-seq analysis was published before on GitHub repository (https://github.com/jakubmie/GCR/blob/main/RNAseq.R).

Statistical analysis

Statistical analysis was carried out using the GraphPad Prism v5 ((https://www.graphpad.com/). Data distributions were assessed for normality using d'Agostino and Pearson omnibus normality tests. For normally distributed datasets, two sample t test, 1-way ANOVA, with Dunn's and Bonferroni's post tests were used to assess statistical significance as appropriate; for non-normally distributed datasets, Mann-Whitney rank test, Kruskal-Wallis test and Dunn's post test were used. Datasets were presented as scatter dot plots with lines at median and cumulative probability plots as appropriate. All the experiments were performed in at least 3 independent experiments.

Supplementary tables

Table S1. Differentially expressed genes following 1 hour fluvoxamine treatment

Gene ID	Protein	logFC	logCPM	PValue	FDR
0 vs 80nM					
ENSRNOG00000019422	Egr1	-1.262908	5.970977	9.172503e-15	1.08951e-10
0 vs 400nM					
ENSRNOG00000030700	Mtco3	0.7164274	12.078370	1.463014e-05	0.04344420
ENSRNOG00000020389	Capn12	0.7803232	6.947520	1.332586e-05	0.04344420
ENSRNOG00000033615	mt-Nd3	1.3063737	8.433068	4.588197e-06	0.02886054
ENSRNOG00000038478	Similar to to Hmg1	1.4902507	3.351332	4.859495e-06	0.02886054
0 vs 2uM					
ENSRNOG00000019422	Egr1	-1.387474	5.970977	2.19506e-17	2.607292e-13

Table S2. Selected physico-chemical properties of ADs used in this study.

Drug	Effect on 4 kDa dextran uptake	Polar Surface Area, Å²	Geom shape coeff	LogP	pKa	Binding to sigma-1 receptor, nM
fluoxetine	neg	21.26	9.79	4.05	9.8	191
citalopram	neg	36.26	10.26	3.76	9.78	404
escitalopram	neg	36.26	9.97	1.34	9.5	288
paroxetine	neg	39.72	14.58	2.53	9.90	2041
sertraline	neg	12.03	9.11	5.51	9.16	31

fluvoxamine	pos	56.84	9.42	3.2	8.86	17
mirtazapine	pos	19.37	8.11	2.9	7.7	>10000
duloxetine	pos	21.26	10.03	4.72	9.7	3533
imipramine	pos	6.48	9.01	4.80	9.4	520
vortioxetine	pos	40.6	n/a	4.76	8.85	1.6

Table S3. Antibodies used in this study

Antigen	Conjugation	Species	Manufacturer	Cat. No.	Dilution
EEA1	-	rabbit	Abcam	ab2900	1/300
LAMP1	-	mouse	Invitrogen	MA1-164	1/300
Rabbit IgG	AlexaFluor488	goat	Bioss	bs-0295G-AF488	1/500
Mouse IgG	AlexaFluor594	goat	SolarBio	K1031G-AF594	1/500

Supplementary Figure Legends

Figure S1. Supplementary Data for Figure 1

(**A**) Quantification of 4 kDa FITC-dextran internalization in PC12 cells treated with fluvoxamine, measured as mean fluorescence signal per cell.

(**B**) Ditto for fluoxetine.

(**C**) Quantification of cell numbers per image in control vs fluvoxamine-treated cultures

(**D**) Ditto for control vs fluoxetine-treated cultures.

(**E**) Quantification of 4 kDa FITC-dextran internalization in PC12 cells treated with vortioxetine, measured as mean fluorescence signal per cell.

(**F**) Ditto for duloxetine.

(**G**) Ditto for imipramine.

(**H**) Ditto for mirtazapine.

(**I**) Ditto for escitalopram.

(**J**) Ditto for sertraline.

(**K**) Ditto for paroxetine.

(**L**) Ditto for citalopram.

(**M**) Representative images of Lucifer yellow internalization in PC12 cells treated with fluvoxamine for 1 h.

(**N**) Quantification of Lucifer yellow endocytosis.

For all panels: ****P<0.0001, ***P<0.001, **P<0.01,*P<0.05, ns - not significant, Kruskal-Wallis test with Dunn's post test.

Figure S2. Supplementary Data for Figure 2

(**A**) Representative images for PC12 cells treated with fluoxetine and immunostained for EEA1 and LAMP1.

(**B**) Quantification of EEA1 levels in fluoxetine-treated cells. ****P<0.0001, Kruskal-Wallis test.**P<0.01, Dunn's multiple comparisons test.

(**C**) Quantification of LAMP1 levels in fluoxetine-treated cells. ****P<0.0001, Kruskal-Wallis test.**P<0.01, Dunn's multiple comparisons test.

(**D**) Representative images of 70 kDa TRITC-dextran internalization in cells treated with fluvoxamine for 1 h.

(**E**) Quantification of 70 kDa TRITC-Dextran internalization. ****P<0.0001, Kruskal-Wallis test.****P<0.0001, Dunn's multiple comparisons test.

(**F**) Representative images of 2 MDa FITC-dextran internalization in PC12 cells treated with fluvoxamine for 1 h.

(**G**) Quantification of 2 MDa FITC-dextran internalization. ****P<0.0001, Kruskal-Wallis test,****P<0.0001, Dunn's multiple comparisons test.

(**H**) Representative images of PC12 cells incubated with 25 μM EIPA and 400nM fluvoxamine for 1 h.

(**I**) Quantification of 4 kDa FITC-Dextran internalization in fluvoxamine- and EIPA-treated cells.****P<0.0001, Kruskal-Wallis test.****P<0.0001,***P<0.001, ns - not significant, Dunn’s multiple comparisons test.

The above experiments were all from three independent experiments, n=30, Scale bar, 5 μm.

Figure S3. Supplementary data for Figure 3.

(**A**) Principal component analysis of all replicates, Dimensions 2-3.

(**B**) Cluster dendrogram for normalized counts generated using *hclust* function with the complete linkage method to find similar clusters. In a hierarchical cluster tree, any two objects in the original data set are eventually linked together at some level. The height of the link represents the distance between the two clusters that contain those two objects.

Figure S4. Supplementary data for Figure 4.

(**A**) Effect of fluvoxamine on the impedance of human brain endothelial cells monitored by real-time measurements for 1 hour. Values presented are means ± SD. Statistical analysis: one-way ANOVA followed by Dunnett's post-test; C, control, TX-100, Triton X-100; n = 6–8.

(**B**) Effect of fluvoxamine on the impedance of human brain endothelial cells monitored by real-time measurements at the 4 h time-point. Values presented are means ± SD. Statistical analysis: one-way ANOVA followed by Dunnett's post-test; C, control, TX-100, Triton X-100; n = 6–8.

(**C**) Cellular uptake of Lucifer yellow alone and in the presence of 80 nM fluvoxamine in cultured human brain endothelial cells after 1 and 4 h of incubation. Values are means ± SD. Statistical analysis: two-way ANOVA, Bonferroni posttest; ***P < 0.001, compared to the control group at a given time-point n = 6. C, control; F, fluvoxamine.

(**D**) Cellular uptake of Atto488-labelled galectin-1 alone and in the presence of 80 nM fluvoxamine in cultured human brain endothelial cells after 1 and 4 h of incubation. Values presented are means ± SD Statistical analysis: two-way ANOVA, Bonferroni posttest, n = 6.

(**E**) Permeability of galectin-1-Atto488 across the human co-culture model of the blood-brain barrier in the presence of 80 nM fluvoxamine after 1, 2 and 4 h of incubation. Values presented are means ± SD. Statistical analysis: two-way ANOVA, Bonferroni posttest; $^{##}$P < 0.01, $^{#}$P < 0.05 difference between groups at a given time-point; *P < 0.05 compared to the control group in each time-points; n = 4.

(**F**) Permeability of Evans- blue labelled albumin (EBA) across the human co-culture model of the blood-brain barrier in the presence of 80 nM fluvoxamine after 1, 2 and 4 h of incubation. Values presented are means ± SD. Statistical analysis: two-way ANOVA, Bonferroni posttest; n = 4.

(**G**) Quantification of FITC signal in brain cortex sections from mice injected intravenously 1 h prior with 4 kDa FITC-dextran and various concentrations of fluvoxamine. *P<0.05, Kruskal-Wallis test with Dunn's post test.

(**H**) Quantification of FITC signal in various internal organs of mice injected intraperitoneally 24 h prior with 4 kDa FITC-dextran and 400 nM fluvoxamine. *P<0.05, Kruskal-Wallis test with Dunn's post test.

Figure S1

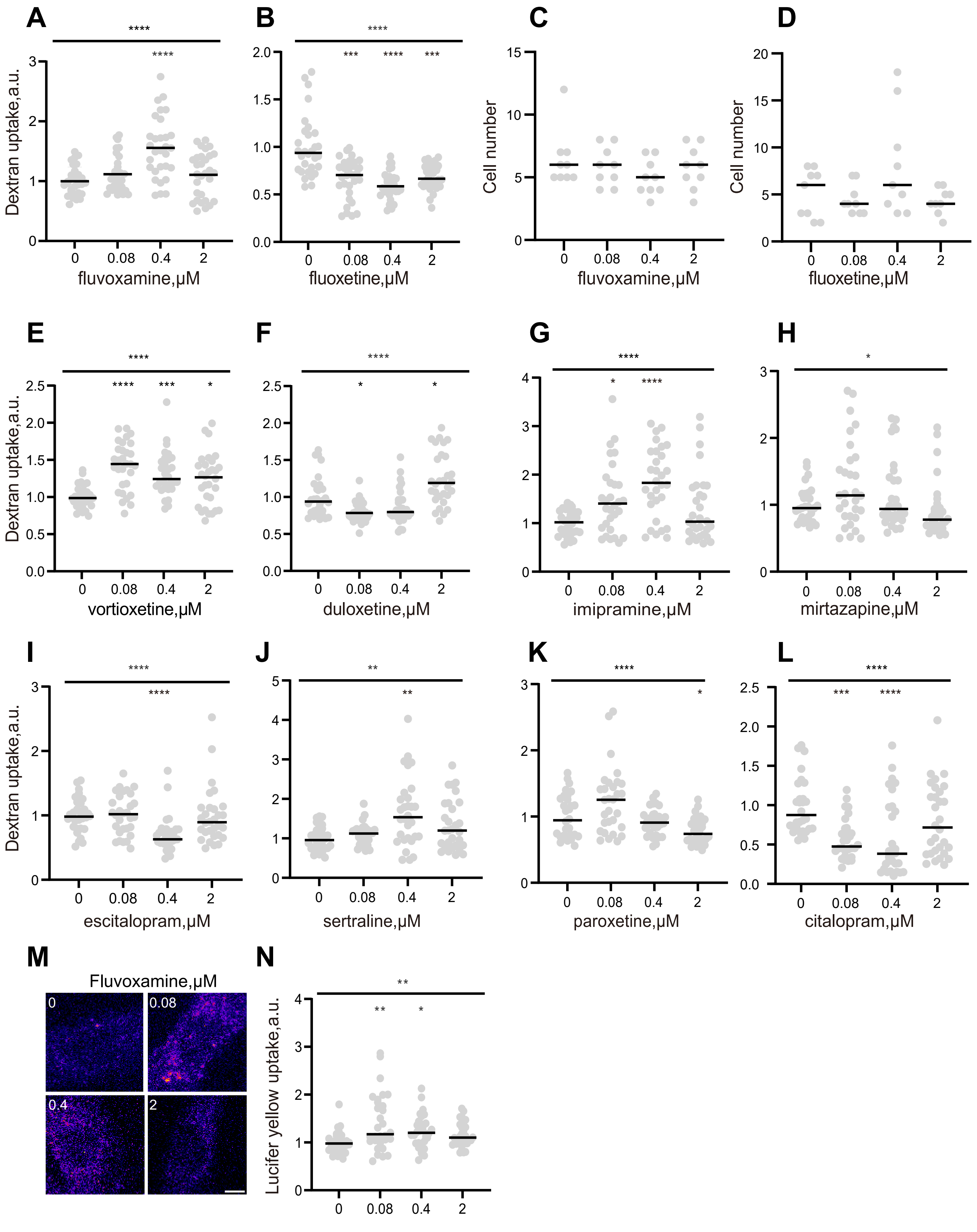

A
B
C
D
Dextran uptake,a.u.
fluvoxamine,μM
fluoxetine,μM
Cell number
E
F
G
H
vortioxetine,μM
duloxetine,μM
imipramine,μM
mirtazapine,μM
I
J
K
L
escitalopram,μM
sertraline,μM
paroxetine,μM
citalopram,μM
M
N
Fluvoxamine,μM
Lucifer yellow uptake,a.u.
0
0.08
0.4
2

Figure S2

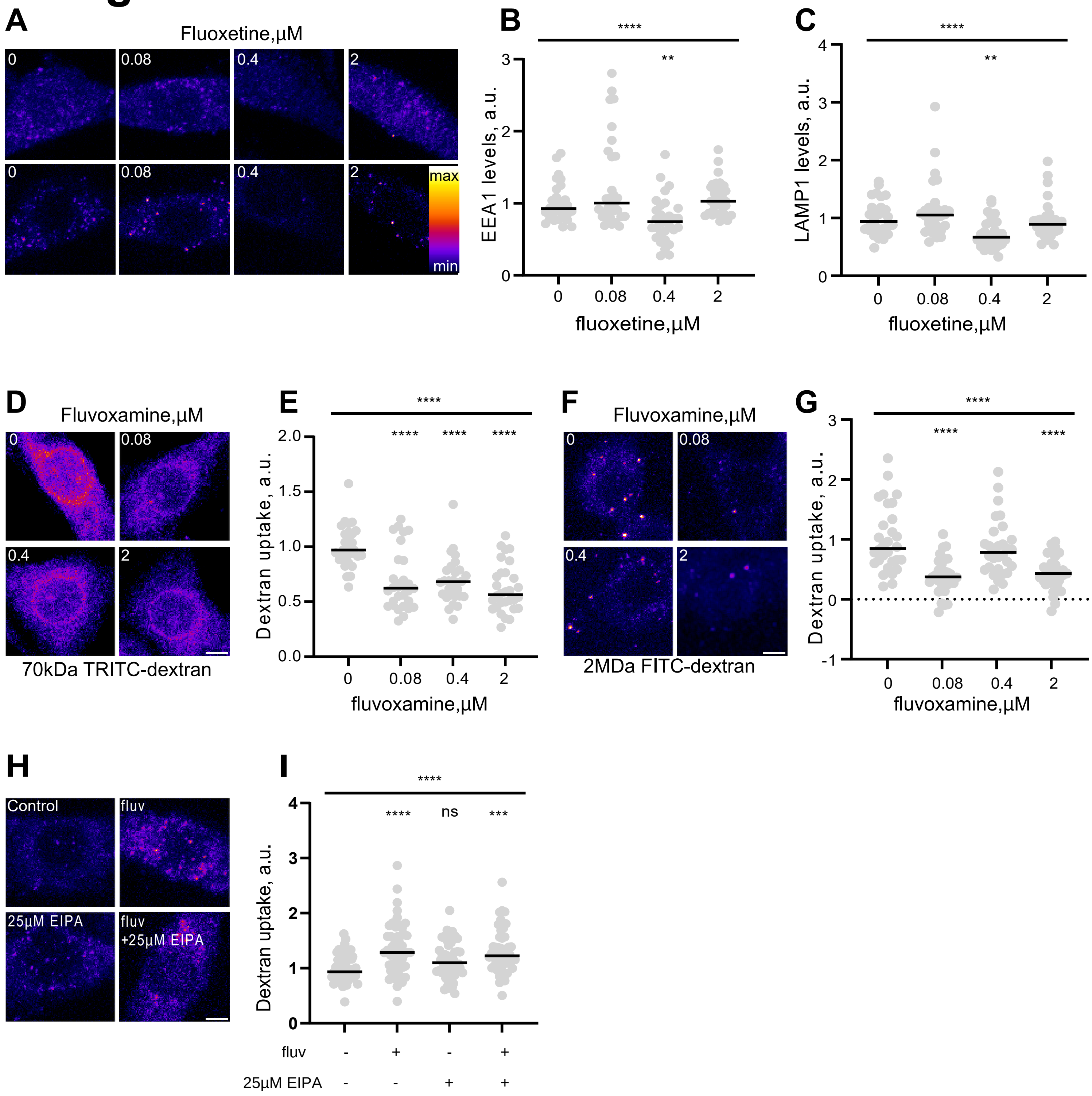
A
Fluoxetine,µM
EEA1
LAMP1
0
0.08
0.4
2
max
min
B
EEA1 levels, a.u.
fluoxetine,µM
C
LAMP1 levels, a.u.
fluoxetine,µM
D
Fluvoxamine,µM
70kDa TRITC-dextran
E
Dextran uptake, a.u.
fluvoxamine,µM
F
Fluvoxamine,µM
2MDa FITC-dextran
G
Dextran uptake, a.u.
fluvoxamine,µM
H
Control
fluv
25µM EIPA
fluv +25µM EIPA
I
Dextran uptake, a.u.
ns
fluv
25µM EIPA

Figure S3

A

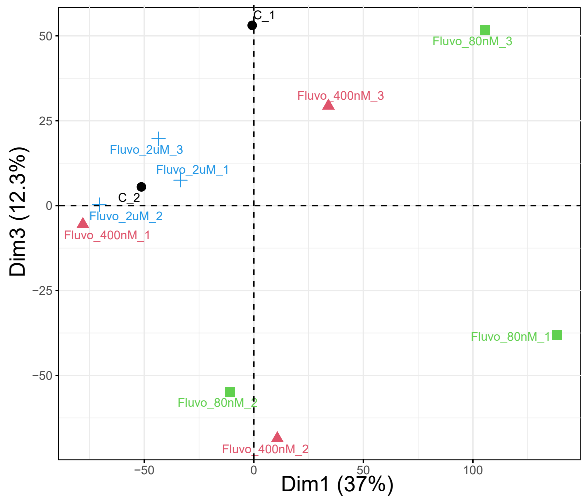
C_1
Fluvo_80nM_3
Fluvo_400nM_3
Fluvo_2uM_3
Fluvo_2uM_1
C_2
Fluvo_2uM_2
Fluvo_400nM_1
Fluvo_80nM_1
Fluvo_80nM_2
Fluvo_400nM_2
Dim3 (12.3%)
Dim1 (37%)

B

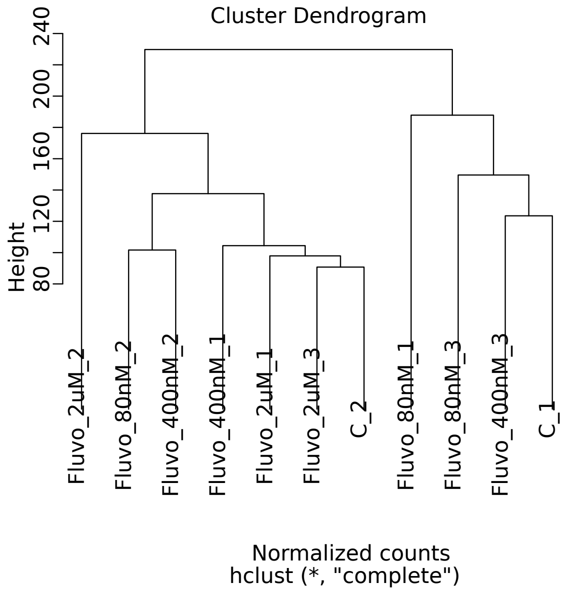
Cluster Dendrogram
Height
Fluvo_2uM_2
Fluvo_80nM_2
Fluvo_400nM_2
Fluvo_400nM_1
Fluvo_2uM_1
Fluvo_2uM_3
C_2
Fluvo_80nM_1
Fluvo_80nM_3
Fluvo_400nM_3
C_1
Normalized counts
hclust (*, "complete")

Figure S4

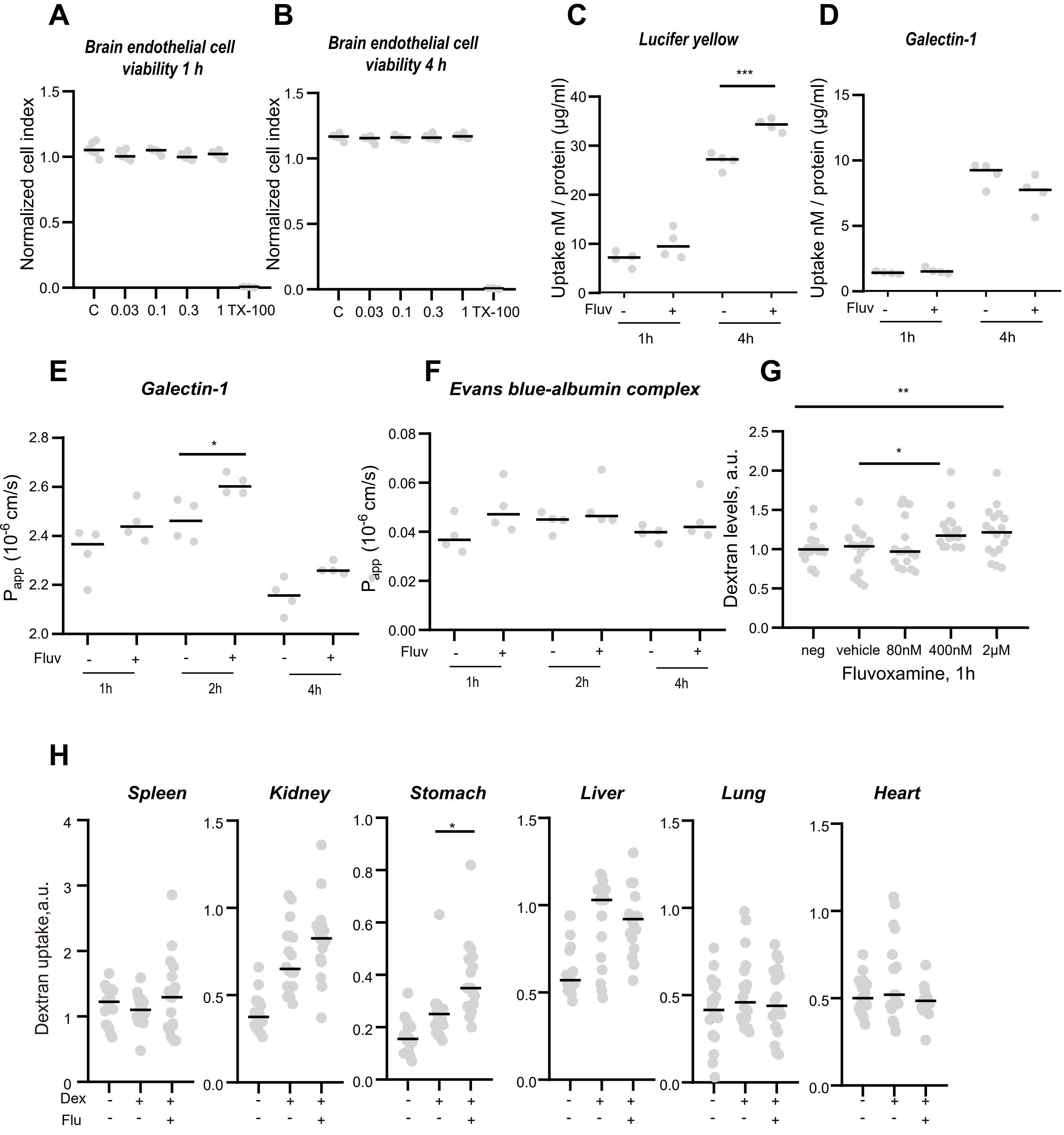

A
Brain endothelial cell viability 1 h
Normalized cell index
1.5
1.0
0.5
0.0
C
0.03
0.1
0.3
1
TX-100
B
Brain endothelial cell viability 4 h
Normalized cell index
C
0.03
0.1
0.3
1
TX-100
C
Lucifer yellow
Uptake nM / protein (μg/ml)
40
30
20
10
0
***
Fluv
-
+
1h
4h
D
Galectin-1
Uptake nM / protein (μg/ml)
15
10
5
0
E
Galectin-1
P_{app} (10^{-6} cm/s)
2.8
2.6
2.4
2.2
2.0
*
Fluv
1h
2h
4h
F
Evans blue-albumin complex
P_{app} (10^{-6} cm/s)
0.08
0.06
0.04
0.02
0.00
G
**
*
Dextran levels, a.u.
2.5
2.0
1.5
1.0
0.5
0.0
neg
vehicle
80nM
400nM
2μM
Fluvoxamine, 1h
H
Spleen
Kidney
Stomach
Liver
Lung
Heart
Dextran uptake,a.u.
Dex
Flu
